# Clinicopathological and Molecular Features of Colorectal Cancer Patients With Mucinous and Non-Mucinous Adenocarcinoma

**DOI:** 10.3389/fonc.2021.620146

**Published:** 2021-03-02

**Authors:** Yuan-Tzu Lan, Shih-Ching Chang, Pei-Ching Lin, Chun-Chi Lin, Hung-Hsin Lin, Shen-Chieh Huang, Chien-Hsing Lin, Wen-Yi Liang, Wei-Shone Chen, Jeng-Kai Jiang, Jen-Kou Lin, Shung-Haur Yang

**Affiliations:** ^1^ Division of Colon and Rectal Surgery, Department of Surgery, Taipei Veterans General Hospital, Taipei, Taiwan; ^2^ Department of Surgery, Faculty of Medicine, School of Medicine, National Yang Ming Chiao Tung University, Taipei, Taiwan; ^3^ Department of Clinical Pathology, Taipei City Hospital, Taipei, Taiwan; ^4^ Department of Health and Welfare, University of Taipei, Taipei, Taiwan; ^5^ Division of Genomic Medicine, National Health Research Institutes, Zhunan, Taiwan; ^6^ Department of Pathology, Taipei Veterans General Hospital, Taipei, Taiwan; ^7^ Department of Surgery, National Yang Ming Chiao Tung University Hospital, Yilan, Taiwan

**Keywords:** colorectal cancer, MAC, NMAC, prognostic factor, genetic alteration

## Abstract

**Background:**

The prognosis of mucinous adenocarcinoma (MAC) and non-mucinous adenocarcinoma (NMAC) in colorectal cancer (CRC) is controversial, and the molecular differences between them are unclear.

**Methods:**

Between 2000 and 2010, a total of 1,483 CRC patients were included. Among them, 73 patients (4.9%) were diagnosed with MAC. The clinicopathological features and genetic alterations were compared between MAC and NMAC.

**Results:**

After propensity score matching to balance age and sex between MAC and NMAC patients, 292 CRC patients (73 MAC and 219 NMAC) were enrolled in the analysis at a 1:3 ratio. In right-sided colon cancer, patients with MAC were more likely to have Borrmann types 3 and 4 tumors, poor differentiation, and advanced T category and tumor, node, metastasis (TNM) stage, chemotherapy, and a similar 5-year overall survival (OS) rate compared with patients with NMAC. In left-sided colon cancer and rectal cancer, patients with MAC were more likely to have Borrmann types 3 and 4 tumors, poor differentiation, lymphovascular invasion, advanced T and N categories and TNM stages, chemotherapy, and a worse 5-year OS rate than patients with NMAC. Regarding genetic alterations, for NMAC, right-sided colon cancer had more *BRAF* mutations than left-sided colon cancer and rectal cancer. For MAC, right-sided colon cancer was associated with more microsatellite instability-high tumors and more *AKT1* mutations than left-sided colon cancer and rectal cancer.

**Conclusion:**

The genetic alterations are distinct between MAC and NMAC in CRC. Tumor location may have an impact on genetic alterations and patient prognosis in MAC and NMAC.

## Introduction

In Taiwan, colorectal cancer (CRC) is the most common type of cancer and the 3^rd^ leading cause of cancer death (1). According to the World Health Organization (WHO) classification, mucinous adenocarcinoma (MAC) of the colon is defined as a tumor in which more than 50% of the extracellular space is occupied by mucin. MAC accounts for 10–15% of CRC (2, 3).

Regarding the clinicopathological and genetic features, MAC was more likely to be located in the proximal colon, to have a microsatellite instability (MSI) phenotype, to have altered expression of *MLH1*, *FHIT*, and *p27* and to have a lower rate of *TP53* mutation than non-mucinous adenocarcinoma (NMAC) (4, 5). The prognosis of MAC compared to NMAC in CRC is debated. Some studies reported that MAC was associated with a worse survival than NMAC (6, 7); however, MAC was reported to have a similar survival (5) and an even better prognosis (8) than NMAC.

This study aimed to compare the difference in clinicopathological and molecular features between MAC and NMAC in CRC patients.

## Materials and Methods

### Patients and Sample Collection

Between 2000 and 2010, a total of 1,483 patients diagnosed as CRC and with available samples were included in this study, and the molecular and clinicopathological features were collected. Among the 1,483 CRC patients, 73 patients were diagnosed as MAC according to the definition of WHO. Written informed consent for sample collection was obtained from all patients. Samples were meticulously dissected and collected from different quadrants of the tumors, snap-frozen in liquid nitrogen, and stored at the Taipei Veterans General Hospital Biobank. The study was approved by the Institutional Review Board of Taipei Veterans General Hospital (Number: 2017-06-004BC), and samples were obtained from the Biobank.

The exclusion criteria included patients who died within 30 days of surgery, received preoperative chemoradiotherapy, underwent emergent operations, non-proven adenocarcinoma, signet ring cell type, recurrent or metachronous cancer. In addition, cancer that occurred in the colon, starting from the cecum to the splenic flexure colon, was defined as right-sided colon cancer. Meanwhile, cancer that occurred in the colon extending from the splenic flexure to the sigmoid colon was considered left-sided colon cancer.

After surgery, patients were followed up every 3 months for the first 2 years and semiannually thereafter. The follow-up protocol included a physical examination, digital rectal examination, carcinoembryonic antigen analysis, chest radiography, abdominal sonogram, and computerized tomography if needed. According to the treatment guideline of colorectal cancer in our hospital, colonoscopy was performed one year after the operation and repeated in one year or 3 years based on the presence or absence of advanced adenoma. Proton emission tomography or magnetic resonance imaging was arranged for patients with elevated levels of carcinoembryonic antigen with an unknown site of tumor recurrence.

Patients received curative surgery followed by adjuvant chemotherapy with 5-FU-based regimen or FOLFOX (folinic acid, fluorouracil, and oxaliplatin). Patients with unresectable metastasis or recurrent disease received palliative chemotherapy with FOLFIRI (folinic acid, fluorouracil, and irinotecan) or FOLFOX. Targeted therapies such as bevacizumab, cetuximab, and panitumumab were not reimbursed by the Taiwan National Health Insurance Administration before 2010.

### DNA Extraction and Analysis of Genetic Mutations

DNA was extracted using a QIAamp DNA Tissue Kit (Qiagen, Valencia, CA, USA) according to the manufacturer’s recommendations. The DNA quality was confirmed using a Nanodrop 1000 Spectrophotometer (Thermo Fisher Scientific). A 12-gene panel with the identification of 139 mutations from selected hotspots was investigated in the Caralogue of Somatic Mutations in Cancer (COSMIC) database and previous studies (9, 10). As described in a previous study (11), the MassArray method was used to detect the mutations of the 139 hotspots in 12 genes.

### MSI Phenotype Analysis

According to international criteria (12), five reference microsatellite markers, which included D5S345, D2S123, BAT25, BAT26, and D17S250 were used to determine the MSI phenotype. Samples with ≥2 positive MSI markers were defined as MSI-high, and those with 0–1 positive MSI markers were defined as microsatellite stable (MSS).

### Propensity Score Matching Strategy

As shown in **Table 1**, to minimize selection bias, propensity score matching was performed based on logistic regression modeling for two covariates (age and sex) to balance potential confounders between males and females. A 1:3 ratio was applied to match MAC and NMAC. A specific caliper width equal to 0.1 standard deviation was used.

**Table 1:** Clinicopathological features of MAC and NMAC in CRC.

	Before propensity-score matching			After propensity-score matching		
	NMAC n = 1410 n (%)	MAC n = 73 n (%)	*P* value	NMAC n = 219 n (%)	MAC n = 73 n (%)	*P* value
Age (years)			0.291			1.000
<70	610 (43.3)	27 (37.0)		81 (37.0)	27 (37.0)	
≥70	800 (56.7)	46 (63.0)		138 (63.0)	46 (63.0)	
Gender			0.074			1.000
Male	919 (65.2)	55 (75.3)		165 (75.3)	55 (75.3)	
Female	491 (34.8)	18 (24.7)		54 (24.7)	18 (24.7)	
Tumor location			**0.002**			**<0.001**
Right-sided colon	360 (25.5)	32 (43.8)		34 (15.5)	32 (43.8)	
Left-sided colon	512 (36.3)	21 (28.8)		80 (36.5)	21 (28.8)	
Rectum	538 (38.2)	20 (27.4)		105 (48.0)	20 (27.4)	
Tumor differentiation			**<0.001**			**<0.001**
Well to moderate	1340 (95.0)	55 (75.3)		217 (99.1)	55 (75.3)	
Poor	70 (5.0)	18 (24.7)		2 (0.9)	18 (24.7)	
Lymphovascular invasion			**0.009**			**<0.001**
Absent	1142 (81.0)	50 (68.5)		207 (94.5)	50 (68.5)	
Present	268 (19.0)	23 (31.5)		12 (5.5)	23 (31.5)	
Pathological T category			**<0.001**			**<0.001**
T1	52 (3.7)	1 (1.4)		52 (23.7)	1 (1.4)	
T2	175 (12.4)	3 (4.1)		142 (64.8)	3 (4.1)	
T3	1037 (73.5)	46 (63.0)		25 (11.4)	46 (63.0)	
T4	146 (10.4)	23 (31.5)		0	23 (31.5)	
Pathological N category			0.068			**<0.001**
N0	772 (54.8)	31 (42.5)		194 (88.6)	31 (42.5)	
N1	331 (23.5)	25 (34.2)		19 (8.7)	25 (34.2)	
N2	307 (21.8)	17 (23.3)		6 (2.7)	17 (23.3)	
Pathological TNM stage			**0.008**			**<0.001**
I	189 (13.4)	1 (1.4)		169 (77.2)	1 (1.4)	
II	539 (38.2)	25 (34.2)		24 (11.0)	25 (34.2)	
III	443 (31.4)	30 (41.1)		24 (11.0)	30 (41.4)	
IV	239 (17.0)	17 (23.3)		2 (0.9)	17 (23.3)	
MSI status			0.078			0.140
MSI-H	141 (10.0)	12 (16.4)		22 (10.0)	12 (16.4)	
MSS	1269 (90.0)	61 (83.6)		197 (90.0)	61 (83.6)	
Chemotherapy			**0.001**			**<0.001**
No	760 (53.9)	25 (34.2)		188 (85.8)	25 (34.2)	
Yes	650 (46.1)	48 (65.8)		31 (14.2)	48 (65.8)	

CRC, colorectal cancer; NMAC, non-mucinous adenocarcinoma; MAC, mucinous adenocarcinoma; MSI, microsatellite instability; MSS, microsatellite stable; TNM, tumor, node, metastasis; bold, statistically significant.

### Statistical Analysis

Statistical analyses were performed using IBM SPSS Statistics 25.0 (IBM Corp., Armonk, NY, USA). The statistical endpoint for overall survival (OS) was measured from the date of surgery until the date that the patient died from any cause. Kaplan–Meier survival curves were plotted and compared using the log-rank test. The impact of the molecular and clinicopathological features on OS was assessed using univariate and multivariate Cox regression analyses. Chi-squared and two-tailed Fisher’s exact tests were used to compare the clinicopathological features. Numerical values were compared using Student’s t-test. Statistical significance was defined as p < 0.05.

## Results

### Clinical Data

Among the 1,483 CRC patients, 73 patients (4.9%) were diagnosed with MAC. As shown in **Table 1**, patients with MAC were more likely to have right-sided colon tumors, poorer differentiation, more lymphovascular invasion, more T4 tumors, more advanced tumor, node, metastasis (TNM) stage, and more chemotherapy than patients with NMAC.

As shown in **Table 1**, after propensity score matching of two covariates (age and sex) with a 1:3 ratio, 292 patients (73 MAC and 219 NMAC) were included in the subsequent analysis. Among the 292 patients, 19 patients were stage IV diseases, and palliative resection was performed. The other 273 patients received curative resection (R0), and none of them received R1 or R2 resection. Patients with MAC were more likely to have Borrmann type 3 and 4 tumors, right-sided colon tumors, poor differentiation, and more advanced T and N categories and TNM stage, and more chemotherapy than patients with NMAC.

As shown in **Table 2**, for right-sided colon cancer, patients with MAC were more likely to have Borrmann types 3 and 4 tumors, poor differentiation, an advanced pathological T category and TNM stage, and chemotherapy than patients with NMAC. For left-sided colon cancer, patients with MAC were more likely to have Borrmann types 3 and 4 tumors, poor differentiation, lymphovascular invasion, advanced pathological T and N categories and TNM stages, and more chemotherapy than patients with NMAC. For rectal cancer, patients with MAC were more likely to have Borrmann types 3 and 4 tumors, poor differentiation, lymphovascular invasion, advanced pathological T and N categories and TNM stages, and more chemotherapy than patients with NMAC.

**Table 2:** Clinicopathological features of MAC and NMAC in CRC stratified by tumor location.

	Right-sided colon cancer			Left-sided colon cancer			Rectal cancer		
	NMAC n = 34 n (%)	MAC n = 32 n (%)	*P* value	NMAC n = 80 n (%)	MAC n = 21 n (%)	*P* value	NMAC n = 105 n (%)	MAC n = 20 n (%)	*P* value
Age (years)			0.141			0.813			0.419
<70	9 (26.5)	14 (43.8)		25 (31.3)	6 (28.6)		47 (44.8)	7 (35.0)	
≥70	25 (73.5)	18 (56.3)		55 (68.8)	15 (71.4)		58 (55.2)	13 (65.0)	
Gender			0.862			0.337			0.069
Male	23 (67.6)	21 (65.6)		68 (85.0)	16 (76.2)		74 (70.5)	18 90.0()	
Female	11 (32.4)	11 (34.4)		12 (15.0)	5 (23.8)		31 (29.5)	2 (10.0)	
Type of operation			0.913			0.736			0.622
Right hemicolectomy	28 (82.4)	26 (81.3)		0	0		0	0	
Extended right hemicolectomy	5 (14.7)	5 (15.6)		0	0		0	0	
Transverse colectomy	1 (2.9)	1 (3.1)		0	0		0	0	
Left hemicolectomy	0	0		15 (18.8)	5 (23.8)		0	0	
Anterior resection	0	0		58 (72.5)	13 (61.9)		5 (4.8)	4 (20.0)	
Low anterior resection	0	0		7 (8.8)	3 (14.3)		92 (87.6)	13 (65.0)	
Abdominoperineal resection	0	0		0	0		8 (7.6)	3 (15.0)	
Tumor differentiation			**0.001**			**0.001**			**<0.001**
Well to moderate	34 (100)	23 (71.9)		78 (97.5)	16 (76.2)		105 (100)	16 (80.0)	
Poor	0	9 (28.1)		2 (2.5)	5 (23.8)		0	4 (20.0)	
Lymphovascular invasion			0.271			**<0.001**			**<0.001**
Absent	30 (88.2)	25 (78.1)		77 (96.3)	13 (61.9)		100 (95.2)	12 (60.0)	
Present	4 (11.8)	7 (21.9)		3 (3.8)	8 (38.1)		5 (4.8)	8 (40.0)	
Pathological T category			**<0.001**			**<0.001**			**<0.001**
T1	11 (32.4)	0		18 (22.5)	0		23 (21.9)	1 (5.0)	
T2	16 (47.1)	2 (6.3)		53 (66.3)	0		73 (69.5)	1 (5.0)	
T3	7 (20.6)	21 (65.6)		9 (11.3)	13 (61.9)		9 (8.6)	12 (60.0)	
T4	0	9 (28.1)		0	8 (38.1)		0	6 (30.0)	
Pathological N category			0.050			**<0.001**			**<0.001**
N0	29 (85.3)	18 (56.3)		72 (90.0)	7 (33.3)		93 (88.6)	6 (30.0)	
N1	2 (5.9)	10 (31.3)		8 (10.0)	6 (28.6)		9 (8.6)	9 (45.0)	
N2	3 (8.8)	4 (12.5)		0	8 (38.1)		3 (2.9)	5 (25.0)	
Pathological TNM stage			**<0.001**			**<0.001**			**<0.001**
I	22 (64.7)	0		62 (77.5)	0		85 (81.0)	1 (5.0)	
II	7 (20.6)	16 (50.0)		9 (11.3)	6 (28.6)		8 (7.6)	3 (15.0)	
III	4 (11.8)	12 (37.5)		8 (10.0)	9 (42.9)		12 (11.4)	9 (45.0)	
IV	1 (2.9)	4 (12.5)		1 (1.3)	6 (28.6)		0	7 (35.0)	
MSI status			0.669			0.136			0.130
MSI-H	7 (20.6)	8 (25.0)		4 (5.0)	3 (14.3)		11 (10.5)	0	
MSS	27 (79.4)	24 (75.0)		76 (95.0)	18 (85.7)		94 (89.5)	20 (100)	
Chemotherapy			**<0.001**			**<0.001**			**<0.001**
5-FU-based alone	3 (8.8)	15 (46.9)		9 (11.3)	7 (33.3)		13 (12.4)	6 (30.0)	
5-FU + Oxaliplatin	0	4 (12.5)		3 (3.8)	3 (14.3)		2 (1.9)	4 (20.0)	
5-FU + Irinotican	1 (2.9)	3 (9.4)		0	3 (14.3)		0	3 (15.0)	

CRC, colorectal cancer; NMAC, non-mucinous adenocarcinoma; MAC, mucinous adenocarcinoma; MSI, microsatellite instability; MSS, microsatellite stable; TNM, tumor, node, metastasis; bold, statistically significant.

As shown in **Supplemental Table 1**, for NMAC, patients with left-side colon cancer were more likely to be males than patients with right-sided colon cancer or rectal cancer. For MAC, patients with rectal cancer were more likely to be males and have more advanced pathological TNM stages and fewer MSI-H tumors than patients with right-sided or left-sided colon cancer.

### Molecular Analysis

As shown in **Table 3**, patients with MAC had fewer *TP53* mutations and more mutations in *TGFβ* and *AKT1* than patients with NMAC. In right-sided colon tumors, patients with MAC had fewer *PIK3CA* mutations and more *AKT1* mutations than patients with NMAC. In left-sided colon tumors, patients with MAC had fewer *TP53* mutations than patients with NMAC. In rectal cancer, there was no significant differrence in genetic mutations between MAC and NMAC.

**Table 3:** The mutation spectrum of MAC and NMAC in CRC.

	All CRC patients			Right-sided colon cancer			Left-sided colon cancer			Rectal cancer		
	NMAC n = 219 n (%)	MAC n = 73 n (%)	*P* value	NMAC n = V34 n (%)	MAC n = 32 n (%)	*P* value	NMAC n = 80 n (%)	MAC n = 21 n (%)	*P* value	NMAC n = 105 n (%)	MAC n = 20 n (%)	*P* value
*TP53*	59 (26.9)	11 (15.1)	**0.040**	6 (17.6)	7 (21.9)	0.666	23 (28.7)	1 (4.8)	**0.022**	30 (28.6)	3 (15.0)	0.207
*APC*	64 (29.2)	13 (17.8)	0.055	8 (23.5)	6 (18.8)	0.635	28 (35.0)	4 (19.0)	0.162	28 (26.7)	3 (15.0)	0.268
*PIK3CA*	22 (10.0)	5 (6.8)	0.414	6 (17.6)	0	**0.013**	7 (8.8)	5 (23.8)	0.058	9 (8.6)	0	0.174
*BRAF*	6 (2.7)	4 (5.5)	0.265	4 (11.8)	3 (9.4)	0.753	2 (2.5)	1 (4.8)	0.587	0	0	–
*KRAS*	91 (41.6)	38 (52.1)	0.118	18 (52.9)	20 (62.5)	0.432	26 (32.5)	8 (38.1)	0.629	47 (44.8)	10 (50.0)	0.666
*NRAS*	9 (4.1)	3 (4.1)	1.000	1 (2.9)	1 (3.1)	0.965	2 (2.5)	2 (9.5)	0.142	6 (5.7)	0	0.273
*HRAS*	3 (1.4)	1 (1.4)	1.000	1 (2.9)	1 (3.1)	0.965	1 (1.3)	0	0.607	1 (1.0)	0	0.661
*FBXW7*	22 (10.0)	5 (6.8)	0.414	2 (5.9)	3 (9.4)	0.592	7 (8.8)	1 (4.8)	0.547	13 (12.4)	1 (5.0)	0.337
*PTEN*	2 (0.9)	2 (2.7)	0.245	1 (2.9)	1 (3.1)	0.965	0	0	–	1 (1.0)	1 (5.0)	0.186
*SMAD4*	9 (4.1)	6 (8.2)	0.168	2 (5.9)	3 (9.4)	0.592	5 (6.3)	1 (4.8)	0.797	2 (1.9)	2 (10.0)	0.059
*TGFβ*	5 (2.6)	7 (9.6)	**0.006**	2 (5.9)	5 (15.6)	0.199	1 (1.3)	0	0.607	2 (1.9)	2 (10.0)	0.059
*AKT1*	2 (0.9)	4 (5.5)	**0.017**	0	4 (12.5)	**0.033**	1 (1.3)	0	0.607	1 (1.0)	0	0.661

CRC, colorectal cancer; NMAC, non-mucinous adenocarcinoma; MAC, mucinous adenocarcinoma; bold, statistically significant.

As shown in **Supplemental Table 2**, for NMAC, patients with right-sided colon tumors had more *BRAF* mutations than patients with left-sided colon tumors or patients with rectal tumors. For MAC, patients with right-sided colon tumors had more *AKT1* mutations than patients with left-sided colon tumors or patients with rectal tumors.

### Recurrence Patterns

Among the 292 patients, 273 patients (56 MAC and 217 NMAC) with stages I–III tumors were included in the analysis of recurrence patterns. As shown in **Table 4**, patients with MAC were more likely to experience tumor recurrence, especially local and peritoneal recurrence, than patients with NMAC. In right-sided colon tumors, there was no significant difference in the recurrence pattern between MAC and NMAC. In left-sided colon tumors, patients with MAC were more likely to experience tumor recurrence, especially local and peritoneal recurrence, than patients with NMAC. In rectal cancer, patients with MAC were more likely to experience tumor recurrence, especially local recurrence.

**Table 4:** Patterns of initial recurrence after curative surgery.

Recurrence pattern	All CRC patients			Right-sided colon cancer			Left-sided colon cancer			Rectal cancer		
	NMAC n = 217 n (%)	MAC n = 56 n (%)	*P* value	NMAC n = 33 n (%)	MAC n = 28 n (%)	*P* value	NMAC n = 79 n (%)	MAC n = 15 n (%)	*P* value	NMAC n = 105 n (%)	MAC n = 13 n (%)	*P* value
Total recurrence	24 (11.1)	19 (33.9)	**<0.001**	5 (15.2)	6 (21.4)	0.525	7 (8.9)	8 (53.3)	**<0.001**	12 (11.4)	5 (38.5)	**0.009**
Local	2 (0.9)	7 (12.5)	**<0.001**	0	1 (3.6)	0.274	0	2 (13.3)	**0.001**	2 (1.9)	4 (30.8)	**<0.001**
Liver	11 (5.1)	2 (3.6)	0.639	1 (3.0)	(3.6)	0.906	4 (5.1)	1 (6.7)	0.800	6 (5.7)	0	0.376
Lung	10 (4.6)	4 (7.1)	0.443	3 (9.1)	2 (7.1)	0.782	3 (3.8)	2 (13.3)	0.131	4 (3.8)	0	0.474
Peritoneum	1 (0.5)	6 (10.7)	**<0.001**	1 (3.0)	3 (10.7)	0.227	0	3 (20.0)	**<0.001**	0	0	**-**
Bone	2 (0.9)	0	0.471	0	0	–	0	0	–	2 (1.9)	0	0.616
Others	3 (1.4)	5 (8.9)	**0.003**	0	3 (10.7)	0.054	1 (1.3)	1 (6.7)	0.184	2 (1.9)	1 (7.7)	0.211

NMAC, non-mucinous adenocarcinoma; MAC, mucinous adenocarcinoma; bold, statistically significant.

Some patients had more than one initial recurrence pattern.

As shown in **Supplemental Table 3**, for NMAC, there was no significant difference in the recurrence pattern between differrent locations of CRC. For MAC, patients with rectal tumors were more likely to develop local recurrence than patients with right-sided and left-sided colon tumors.

### Survival Analysis

**Figure 1:**
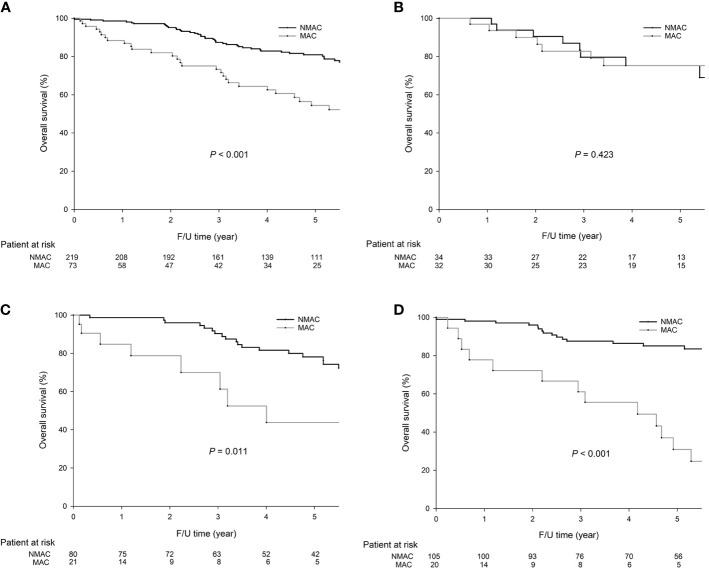
The 5-year overall survival (OS) (54.4 *vs.* 80.9%, *P* = 0.001) rates were significantly lower in mucinous adenocarcinoma (MAC) than in non-mucinous adenocarcinoma (NMAC) in colorectal cancer (CRC). In right-sided colon cancer, the 5-year OS rates were similar between MAC and NMAC (75.3 *vs*. 75.3%, *P* = 0.423). In left-sided colon cancer, the 5-year OS rates were significantly lower in MAC than in NMAC (43.8 *vs*. 78.2%, *P* = 0.011). In rectal cancer, the 5-year OS rates were significantly lower in MAC than in NMAC (30.9 *vs*. 85.1%, *P* < 0.001). The survival curves were as follows: **(A)** OS curves for all 292 CRC patients, **(B)** OS curves for right-sided colon cancer, and **(C)** OS curves for left-sided colon cancer, **(D)** OS curves of rectal cancer.

As shown in **Figure 1**, patients with MAC were associated with significantly worse 5-year OS (59.8 *vs*. 31.1%, *P* < 0.001, **Figure 1A**) than patients with NMAC. In right-sided colon tumors, the 5-year OS rates were similar between MAC and NMAC patients (75.3 *vs*. 75.3%, *P* = 0.423, **Figure 1B**), while in left-sided colon tumors, MAC was associated with a worse 5-year OS than NMAC (43.8 *vs*. 78.2%, *P* = 0.011, **Figure 1C**). In rectal cancer, the 5-year OS rates were significantly lower in MAC than in NMAC (30.9 *vs*. 85.1%, *P* < 0.001, **Figure 1D**).

As shown in **Table 5**, the univariate analysis demonstrated that age, sex, MAC, and pathological TNM stage were significantly associated with OS. The aforementioned four covariates were included in multivariate analysis. Multivariate analysis demonstrated that age and pathological TNM stage were independent prognostic factors affecting OS.

**Table 5:** Univariate and multivariate analysis of overall survival in MAC and NMAC in CRC.

	Univariate analysis			Multivariate analysis		
	Hazard ratio	Confidence interval	*P* value	Hazard ratio	Confidence interval	*P* value
Age (year)			**<0.001**			**0.002**
<70	1.00			1.00		
≥70	2.75	1.688–4.464		2.19	1.568–3.907	
Gender			**0.007**			
Male	1.00					
Female	0.45	0.253–0.807				
Tumor location			0.833			
Right-sided colon	1.00					
Left-sided colon	1.05	0.628–1.749				
Rectum	0.92	0.561–1.507				
Histological type			**0.002**			
NMAC	1.00					
MAC	1.91	1.281–2.859				
Lymphovascular invasion			0.211			
Absent	1.00					
Present	1.47	0.804–2.688				
Pathological TNM stage			**<0.001**			**0.001**
I	1.00			1.00		
II	2.97	1.899–4.642		2.01	1.201–3.357	
III	1.74	1.036–2.907		1.23	0.664–2.265	
IV	5.78	2.685–12.447		4.33	1.838–10.202	
MSI status			0.512			
MSS	1.00					
MSI-H	1.21	0.688–2.118				
Chemotherapy			0.628			
No	1.00					
Yes	1.11	0.732–1.676				

CRC, colorectal cancer; T, tumor; N, node; NMAC, non-mucinous adenocarcinoma; MAC, mucinous adenocarcinoma; MSI, microsatellite instability; MSS, microsatellite stable; bold, statistically significant.

## Discussion

MAC is a distinct form of CRC that is found in 10–15% of CRC patients and is considered an unfavorable subtype of CRC. In the present study, MAC accounted for 4.9% of all CRC cases. MAC is more frequently found in the proximal colon and less common in Asian countries. Whether MAC is associated with prognosis in CRC patients is controversial (5–8). Right-sided colon cancer tends to have a more advanced tumor stage and poor differentiation and be mucinous (9). Interestingly, in the present study, MAC was associated with a worse 5-year OS rate than NMAC only in left-sided colon and rectum. However, multivariate analysis demonstrated that MAC was not an independent prognostic factor. The reason for this finding might be that MAC was diagnosed at a more advanced stage than NMAC. Compared with NMAC, MAC has a less firm consistency, which may cause symptoms to arise only when the tumor reaches an advanced stage. In addition, in the present study, the majority of recurrence in NMAC was liver metastasis, while MAC more frequently had extrahepatic metastases, such as recurrence in the locoregional area and peritoneum, especially in left-sided colon and rectum, which was associated with poor prognosis (10–12). Among the 56 MAC patients who underwent curative resection, 17 patients had extrahepatic metastases. Among the 17 patients, the most frequent mutated gene was *KRAS* (52.9%), followed by *TP53* (29.4%), and *PIK3CA*. Genetic mutations might play a role in extrahepatic metastases in MAC.

According to our results, MAC had a more advanced TNM stage than NMAC. For MAC, rectal cancer was associated with a more advanced TNM stage than right-sided and left-side colon cancer; however, for NMAC, there was no significant difference in the TNM stage between different tumor locations. The above reason might explain a higher risk of tumor recurrence and peritoneal seeding in MAC, especially in rectal cancer.

When MAC is diagnosed with metastatic disease, the prognosis of patients is usually worse than that of patients with metastatic NMAC. The main reason is the poor response of metastatic MAC to chemotherapy, which might be due to a higher frequency of MSI and a higher level of DNA topoisomerase 1 expression in MAC than NMAC (13, 14). According to the National Comprehensive Cancer Network (NCCN) guidelines, MSI testing is recommended for all patients with stage II CRC because patients with MSI-H tumors may have a good prognosis and obtain no benefit from 5-FU-based adjuvant chemotherapy. In addition, the mucous produced by MAC might function as a physical barrier to the delivery of chemotherapy into cancer cells. To date, it is unclear whether the MSI status of MAC has an impact on the treatment response of 5-FU-based chemotherapy in CRC. The enrollment of more MAC patients is required to answer this question. Compared with NMAC, MAC was reported to be associated with different molecular features, such as MSI and mutations in *BRAF*, *KRAS*, and *PIK3CA* (15). In the present study, there was no significant difference in *BRAF*, *KRAS*, and *PIK3CA* mutations between MAC and NMAC in CRC patients. MAC was associated with significantly fewer *PIK3CA* mutations than NMAC in only right-sided colon cancer. In addition, for MAC, right-sided colon cancer had more MSI-H tumors (25.0 *vs*. 19.0 *vs*. 0%, *P*=0.022) and earlier TNM stage than left-sided colon cancer and rectal cancer; for NMAC, there was no significant difference in MSI status and TNM stage between different tumor locations. More advanced tumor stage may explain poor survival in MAC located in the left-sided colon and rectum. MSI-H CRC is a biomarker for a potential response to immunotherapy, and immunotherapy was approved by the FDA for the treatment of metastatic MSI-H CRC. According to our results, we recommend MSI testing for MAC, especially for right-sided colon cancer, which might provide useful information about immunotherapy for MAC.

In the present study, our results demonstrated that MAC was associated with fewer *TP53* mutations than NMAC, especially in left-sided colon cancer. It seems that *TP53* mutations did not play an important role in the carcinogenesis of MAC in left-sided colon cancer. Moreover, our results showed that MAC was associated with more *TGFβ* mutations than NMAC, especially in right-sided colon cancer. *TGFβ* plays an important role in tumor progression, allowing cancer cells to escape immune surveillance, proliferate, invade and metastasize. Inhibiting *TGFβ* can impact regulatory T cell production and potentially augment the effect of PD-1/PD-L1 inhibitors, which can enhance treatment responses (16). In addition, our results showed that patients with MAC had more *AKT1* mutations than patients with NMAC, especially in right-sided colon cancer. *AKT1* activation can induce *PI3K/AKT* pathway activation, which is one of the most frequently activated pathways in cancer (17). AZD5363, a pan-AKT inhibitor, was reported to improve progression-free survival in advanced cancers with *AKT1* mutations (18) However, the number of patients with MAC is too small to draw a conclusion on the pattern of *AKT1*, *PIK3CA*, *TGFβ* mutation. The enrollment of more patients is required to validate our results.

There were some limitations to the present study. First, this was a retrospective study from a single institute, and selection bias could exist. Second, MAC is an uncommon histological type of CRC, and the sample size was small. Third, since the patient number varied greatly between MAC and NMAC, we used propensity score matching to decrease the selection bias. Fourth, although our results demonstrated that there were significant differences in the genetic alterations between MAC and NMAC, in subgroup analysis, some patient numbers were small, and selection bias could have existed.

## Conclusion

The present study demonstrated that MAC was associated with more tumor recurrence and a worse survival than NMAC in left-sided colon and rectum, while no difference was observed between MAC and NMAC in right-sided colon cancer. Although some genetic mutations were distinct between MAC, NMAC, and different tumor locations, the number of MAC patients in the present study were too small to make the conclusion. The enrollment of more patients is required to validate our results.

## Data Availability Statement

The raw data supporting the conclusions of this article will be made available by the authors, without undue reservation.

## Ethics Statement

The studies involving human participants were reviewed and approved by the Institutional Review Board of Taipei Veterans General Hospital. The patients/participants provided their written informed consent to participate in this study.

## Author Contributions

Conceptualization: Y-TL, S-CC, P-CL, C-CL, H-HL, S-CH, C-HL, W-YL, W-SC, J-KJ, J-KL, and S-HY. Data curation: C-HL. Formal analysis, S-CC and Y-TL. Funding acquisition: S-CC. Investigation: C-HL. Methodology: C-HL. Writing—original draft: S-CC and Y-TL. Writing—review and editing: S-CC and Y-TL. All authors contributed to the article and approved the submitted version.

## Funding

This study was supported by the Taipei Veterans General Hospital (V105C-043, V106C-027, V107C-004), Ministry of Science and Technology, Taiwan (105-2314-B-075-010-MY2), and Taipei City Hospital (10601-62-059, 10701-62-050). The funding body did not play role in the design of the study, and collection, analysis, and interpretation of data, and in writing the manuscript.

## Conflict of Interest

The authors declare that the research was conducted in the absence of any commercial or financial relationships that could be construed as a potential conflict of interest.

## Abbreviations

CRC, colorectal cancer; MAC, mucinous adenocarcinoma; MSI, microsatellite instability; MSS, microsatellite stable; NMAC, non-mucinous adenocarcinoma; LVI, lymphovascular invasion; OS, overall survival; PCR, polymerase chain reaction; TNM, tumor, node, metastasis; WHO, World Health Organization.
