# Supplementary material for: Clinicopathological and Molecular Features of Colorectal Cancer Patients With Mucinous and Non-Mucinous Adenocarcinoma
Source: Front Oncol. 2021 Mar 2;11:620146. doi: 10.3389/fonc.2021.620146 (PMC7962409; doi:10.3389/fonc.2021.620146)
Supplement: Supplementary file 1 [file Table_1.docx]

Supplemental Table 1. Clinicopathological features of NMAC and MAC and NMAC according to tumor location.

|  | NMAC | | | |  | MAC | | | |
| --- | --- | --- | --- | --- | --- | --- | --- | --- | --- |
|  | Right-sided colon cancer  n=34  n (%) | Left-sided colon cancer  n=80  n (%) | Rectal cancer  n=105  n (%) | *P* value |  | Right-sided colon cancer  n=32  n (%) | Left-sided colon cancer  n=21  n (%) | Rectal cancer  n=20  n (%) | *P* value |
| Age (years) |  |  |  | 0.065 |  |  |  |  | 0.522 |
| <70 | 9 (26.5) | 25 (31.3) | 47 (44.8) |  |  | 14 (43.8) | 6 (28.6) | 7 (35.0) |  |
| >70 | 25 (73.5) | 55 (68.8) | 58 (55.2) |  |  | 18 (56.3) | 15 (71.4) | 13 (65.0) |  |
| Gender |  |  |  | **0.040** |  |  |  |  | **0.049** |
| Male | 23 (67.6) | 68 (85.0) | 74 (70.5) |  |  | 21 (65.6) | 16 (76.2) | 18 (90.0) |  |
| Female | 11 (32.4) | 12 (15.0) | 31 (29.5) |  |  | 11 (34.4) | 5 (23.8) | 2 (10.0) |  |
| Tumor differentiation |  |  |  | 0.528 |  |  |  |  | 0.506 |
| Well to moderate | 34 (100) | 78 (97.5) | 105 (100) |  |  | 23 (71.9) | 16 (76.2) | 16 (80.0) |  |
| Poor | 0 | 2 (2.5) | 0 |  |  | 9 (28.1) | 5 (23.8) | 4 (20.0) |  |
| Lymphovascular invasion |  |  |  | 0.239 |  |  |  |  | 0.291 |
| Absent | 30 (88.2) | 77 (96.3) | 100 (95.2) |  |  | 25 (78.1) | 13 (61.9) | 12 (60.0) |  |
| Present | 4 (11.8) | 3 (3.8) | 5 (4.8) |  |  | 7 (21.9) | 8 (38.1) | 8 (40.0) |  |
| Pathological T category |  |  |  | 0.842 |  |  |  |  | 0.805 |
| T1 | 11 (32.4) | 18 (22.5) | 23 (21.9) |  |  | 0 | 0 | 1 (5.0) |  |
| T2 | 16 (47.1) | 53 (69.5) | 73 (69.5) |  |  | 2 (6.3) | 0 | 1 (5.0) |  |
| T3 | 7 (20.6) | 9 (8.6) | 9 (8.6) |  |  | 21 (65.6) | 13 (61.9) | 12 (60.0) |  |
| T4 | 0 | 0 | 0 |  |  | 9 (28.1) | 8 (38.1) | 6 (30.0) |  |
| Pathological N category |  |  |  | 0.501 |  |  |  |  | 0.057 |
| N0 | 29 (85.3) | 72 (90.0) | 93 (88.6) |  |  | 18 (56.3) | 7 (33.3) | 6 (30.0) |  |
| N1 | 2 (5.9) | 8 (10.0) | 9 (8.6) |  |  | 10 (31.3) | 6 (28.6) | 9 (45.0) |  |
| N2 | 3 (8.8) | 0 | 3 (2.9) |  |  | 4 (12.5) | 8 (38.1) | 5 (25.0) |  |
| Pathological TNM stage |  |  |  | 0.140 |  |  |  |  | **0.026** |
| I | 22 (64.7) | 62 (77.5) | 85 (81.0) |  |  | 0 | 0 | 1 (5.0) |  |
| II | 7 (20.6) | 9 (11.3) | 8 (7.6) |  |  | 16 (50.0) | 6 (28.6) | 3 (15.0) |  |
| III | 4 (11.8) | 8 (10.0) | 12 (11.4) |  |  | 12 (37.5) | 9 (42.9) | 9 (45.0) |  |
| IV | 1 (2.9) | 1 (1.3) | 0 |  |  | 4 (12.5) | 6 (28.6) | 7 (35.0) |  |
| MSI status |  |  |  | 0.334 |  |  |  |  | **0.022** |
| MSI-H | 7 (20.6) | 4 (5.0) | 11 (10.5) |  |  | 8 (25.0) | 4 (19.0) | 0 |  |
| MSS | 27 (79.4) | 76 (95.0) | 94 (89.5) |  |  | 24 (75.0) | 17 (81.0) | 20 (100) |  |
| Chemotherapy |  |  |  | 0.389 |  |  |  |  | 0.906 |
| 5-FU-based alone | 3 (8.8) | 9 (11.3) | 13 (12.4) |  |  | 15 (46.9) | 7 (33.3) | 6 (30.0) |  |
| 5-FU + Oxaliplatin | 0 | 3 (3.8) | 2 (1.9) |  |  | 4 (12.5) | 3 (14.3) | 4 (20.0) |  |
| 5-FU + Irinotican | 1 (2.9) | 0 | 0 |  |  | 3 (9.4) | 3 (14.3) | 3 (15.0) |  |

CRC: colorectal cancer; NMAC: non-mucinous adenocarcinoma; MAC: mucinous adenocarcinoma; MSI: microsatellite instability; MSS: microsatellite stable; TNM: tumor, node, metastasis; bold: statistically significant
