# Supplementary material for: Clinicopathological and Molecular Features of Colorectal Cancer Patients With Mucinous and Non-Mucinous Adenocarcinoma
Source: Front Oncol. 2021 Mar 2;11:620146. doi: 10.3389/fonc.2021.620146 (PMC7962409; doi:10.3389/fonc.2021.620146)
Supplement: Supplementary file 2 [file Table_2.docx]

Supplemental Table 2. The mutation spectrum of NMAC and MAC according to tumor location.

|  | NMAC | | | |  | MAC | | | |
| --- | --- | --- | --- | --- | --- | --- | --- | --- | --- |
|  | Right-sided colon cancer  n=34  n (%) | Left-sided colon cancer  n=80  n (%) | Rectal cancer  n=105  n (%) | *P* value |  | Right-sided colon cancer  n=32  n (%) | Left-sided colon cancer  n=21  n (%) | Rectal cancer  n=20  n (%) | *P* value |
| *TP53* | 6 (17.6) | 23 (28.7) | 30 (28.6) | 0.413 |  | 7 (21.9) | 1 (4.8) | 3 (15.0) | 0.390 |
| *APC* | 8 (23.5) | 28 (35.0) | 28 (26.7) | 0.340 |  | 6 (18.8) | 4 (19.0) | 3 (15.0) | 0.751 |
| *PIK3CA* | 6 (17.6) | 7 (8.8) | 9 (8.6) | 0.203 |  | 0 | 5 (23.8) | 0 | 0.648 |
| *BRAF* | 4 (11.8) | 2 (2.5) | 0 | **0.001** |  | 3 (9.4) | 1 (4.8) | 0 | 0.148 |
| *KRAS* | 18 (52.9) | 26 (32.5) | 47 (44.8) | 0.084 |  | 20 (62.5) | 8 (38.1) | 10 (50.0) | 0.215 |
| *NRAS* | 1 (2.9) | 2 (2.5) | 6 (5.7) | 0.331 |  | 1 (3.1) | 2 (9.5) | 0 | 0.720 |
| *HRAS* | 1 (2.9) | 1 (1.3) | 1 (1.0) | 0.438 |  | 1 (3.1) | 0 | 0 | 0.313 |
| *FBXW7* | 2 (5.9) | 7 (8.8) | 13 (12.4) | 0.233 |  | 3 (9.4) | 1 (4.8) | 1 (5.0) | 0.513 |
| *PTEN* | 1 (2.9) | 0 | 1 (1.0) | 0.528 |  | 1 (3.1) | 0 | 1 (5.0) | 0.777 |
| *SMAD4* | 2 (5.9) | 5 (6.3) | 2 (1.9) | 0.173 |  | 3 (9.4) | 1 (4.8) | 2 (10.0) | 0.994 |
| *TGFβ* | 2 (5.9) | 1 (1.3) | 2 (1.9) | 0.315 |  | 5 (15.6) | 0 | 2 (10.0) | 0.378 |
| *AKT1* | 0 | 1 (1.3) | 1 (1.0) | 0.732 |  | 4 (12.5) | 0 | 0 | **0.039** |

CRC: colorectal cancer; NMAC: non-mucinous adenocarcinoma; MAC: mucinous adenocarcinoma; bold: statistically significant
