# Supplementary material for: Clinicopathological and Molecular Features of Colorectal Cancer Patients With Mucinous and Non-Mucinous Adenocarcinoma
Source: Front Oncol. 2021 Mar 2;11:620146. doi: 10.3389/fonc.2021.620146 (PMC7962409; doi:10.3389/fonc.2021.620146)
Supplement: Supplementary file 3 [file Table_3.docx]

Supplemental Table 3. Patterns of initial recurrence after curative surgery.

|  | NMAC | | | |  | MAC | | | |
| --- | --- | --- | --- | --- | --- | --- | --- | --- | --- |
| Recurrence pattern | Right-sided colon cancer  n=33  n (%) | Left-sided colon cancer  n=79  n (%) | Rectal cancer  n=105  n (%) | *P* value |  | Right-sided colon cancer  n=28  n (%) | Left-sided colon cancer  n=15  n (%) | Rectal cancer  n=13  n (%) | *P* value |
| Total recurrence | 5 (15.2) | 7 (8.9) | 12 (11.4) | 0.774 |  | 6 (21.4) | 8 (53.3) | 5 (38.5) | 0.159 |
| Local | 0 | 0 | 2 (1.9) | 0.191 |  | 1 (3.6) | 2 (13.3) | 4 (30.8) | **0.016** |
| Liver | 1 (3.0) | 4 (5.1) | 6 (5.7) | 0.565 |  | 1 (3.6) | 1 (6.7) | 0 | 0.683 |
| Lung | 3 (9.1) | 3 (3.8) | 4 (3.8) | 0.302 |  | 2 (7.1) | 2 (13.3) | 0 | 0.557 |
| Peritoneum | 1 (3.0) | 0 | 0 | 0.066 |  | 3 (10.7) | 3 (20.0) | 0 | 0.463 |
| Bone | 0 | 0 | 2 (1.9) | 0.191 |  | 0 | 0 | 0 | - |
| Others | 0 | 1 (1.3) | 2 (1.9) | 0.422 |  | 3 (10.7) | 1 (6.7) | 1 (7.7) | 0.706 |

NMAC: non-mucinous adenocarcinoma; MAC: mucinous adenocarcinoma; bold: statistically significant

Some patients had more than one initial recurrence pattern.
